# The cell biology of suturing tendons

**DOI:** 10.1016/j.matbio.2010.06.002

**Published:** 2010-07

**Authors:** J.K.F. Wong, S. Alyouha, K.E. Kadler, M.W.J. Ferguson, D.A. McGrouther

**Affiliations:** a Plastic Surgery Research, University of Manchester, Stopford Building, Oxford Road, Manchester, M13 9PT, United Kingdom; b Wellcome Trust Centre for Cell-Matrix Research, Faculty of Life Sciences, Michael Smith Building, University of Manchester, Oxford Road, Manchester, M13 9PT, United Kingdom; c Faculty of Life Sciences, University of Manchester, Stopford Building, Oxford Road, Manchester, M13 9PT and Renovo Plc. The Manchester Incubator Building, 48 Grafton Street, Manchester, M13 9XX, United Kingdom; d Plastic Surgery Research, University of Manchester, Stopford Building, Oxford Road, Manchester, M13 9PT, United Kingdom

**Keywords:** ST, Subcutaneous tissue, Tendon, Mouse, Trauma, Suture, Inflammation, Necrosis

## Abstract

Trauma by suturing tendon form areas devoid of cells termed “acellular zones” in the matrix. This study aimed to characterise the cellular insult of suturing and acellular zone formation in mouse tendon. Acellular zone formation was evaluated using single grasping sutures placed using flexor tendons with time lapse cell viability imaging for a period of 12 h. Both tension and injury were required to induce cell death and cell movement in the formation of the acellular zone. DNA fragmentation studies and transmission electron microscopy indicated that cells necrosed.

Parallel *in vivo* studies showed that cell-to-cell contacts were disrupted following grasping by the suture in tensioned tendon. Without tension, cell death was lessened and cell-to-cell contacts remained intact. Quantitative immunohistochemistry and 3D cellular profile mapping of wound healing markers over a one year time course showed that acellular zones arise rapidly and showed no evidence of healing whilst the wound healing response occurred in the surrounding tissues. The acellular zones were also evident in a standard modified “Kessler” clinical repair. In conclusion, the suture repair of injured tendons produces acellular zones, which may potentially cause early tendon failure.

## 1 Introduction

Suturing of organised tissues plays a day-to-day role in repairing damaged structures in a surgeon's armamentarium. However the cellular trauma of suturing has never really been investigated. Suturing involves a number of mechanical insults on tissues and their cells. Suturing is initiated through the insertion of the sharp needle end that can be either a cutting or piercing injury. The cellular insult following this is small resulting in localised injury where the needle and suture pass through the tissue. The second insult is from the tying of the suture and applying a compressive force to the matrix and cells. This insult we have shown in the past to form an “acellular zone” (Wong et al., 2006). Finally the third insult arises from the persistence of a suture as a foreign material and how the systemic processes *in vivo* react to the retained material. Tendon lends itself well to the investigation of suture biology as it is a relatively homogeneous tissue with few specialised cell populations, hence local and systemic cellular responses can be observed with relative ease (Kajikawa et al., 2007). Furthermore it is a tissue with limited vasculature and hence less prone to ischemia and also has a well organised cellular network that allows cells to interact with each other via gap junctions (Ralphs, Waggett et al., 2002). Most importantly it is a tissue that is prone to traumatic insult either through lacerations e.g. in hand trauma (Elliot, 2002), or through rupture from sporting injuries (Ljungqvist, Schwellnus et al., 2008) with considerable financial implications to both healthcare provision and the manual workforce. With either of these major clinical problems the mainstay of treatment is surgical suture repair. In the process of searching for the ideal suture repair technique numerous novel methods have been devised to unite cut tendon ends. These techniques have often been developed on cadaveric human or animal models and assessed via biomechanical means such as testing load to failure, gliding excursion and gap formation (reviewed by Strickland, 2000). In the last decade there has been a trend to increase the number of core strands (Savage, 1985, Strickland, 2000), and the calibre of the suture used in repair (Taras, Raphael et al., 2001). Locking sutures as opposed to grasping methods have been incorporated into the core suture (Hotokezaka and Manske, 1997, Pennington, 1979) and supplemental epitendinous suture with interlocking properties have also been favoured in *ex vivo* studies (Dona, Turner et al., 2003). All of these repairs have the primary emphasis of adding tensile strength (Thurman, Trumble et al., 1998) and minimising gap formation (Gelberman, Boyer et al., 1999) in the repair but lead to increasing amounts of suture material being placed in the tendon substance. The cellular reaction to these multi-stranded techniques is not clear.

Importantly, scientific evidence to demonstrate the damage a surgeon can inflict from even the most trivial surgical procedures, such as a single suture, must be clarified. The possibility that our practice is negatively impacting on tissue biology has fundamental implications to all surgical procedures involving the suturing of tissue. The present study aims to investigate the formation of the acellular zone and the role of cell death in its formation. To understand the mechanisms of acellular formation, we used a combination of surgical manipulation in an explant live/dead time lapse culture system, transmission electron microscopy, *in vivo* immunohistochemical staining, and DNA fragmentation gel analysis. A time course study was performed over one year in a murine model to elucidate the fate of the acellular zone. Furthermore a modified Kessler repair was performed in mouse Achilles tendon to assess the distribution of acellular zones in a clinically relevant repair.

## 2 Results

### 2.1 Role of tension in acellular zone formation

**Fig. 1:**
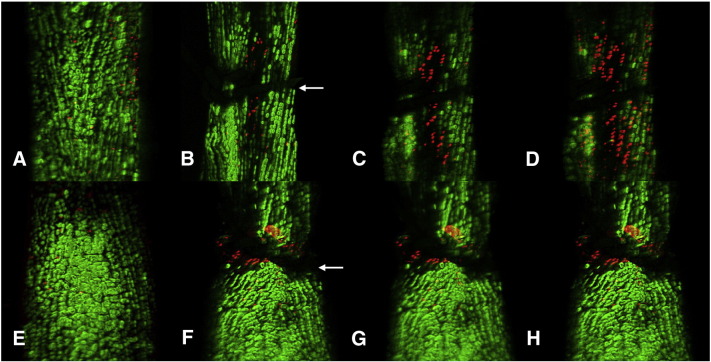
Live/dead assay time lapse confocal microscopy of sutured tendon. Tendon is placed under tension (A, B, C and D) or without tension (E, F, G and H). Imaging of tendon before suturing (A and E). Images taken 30 min after suture (B and F), 6 h following injury (C and G), and 12 h following injury (D and H). Note formation of an acellular zone within 30 min of suturing tendon under tension and propagation of cell death over time compared to un-tensioned sutured tendon.

**Fig. 2:**
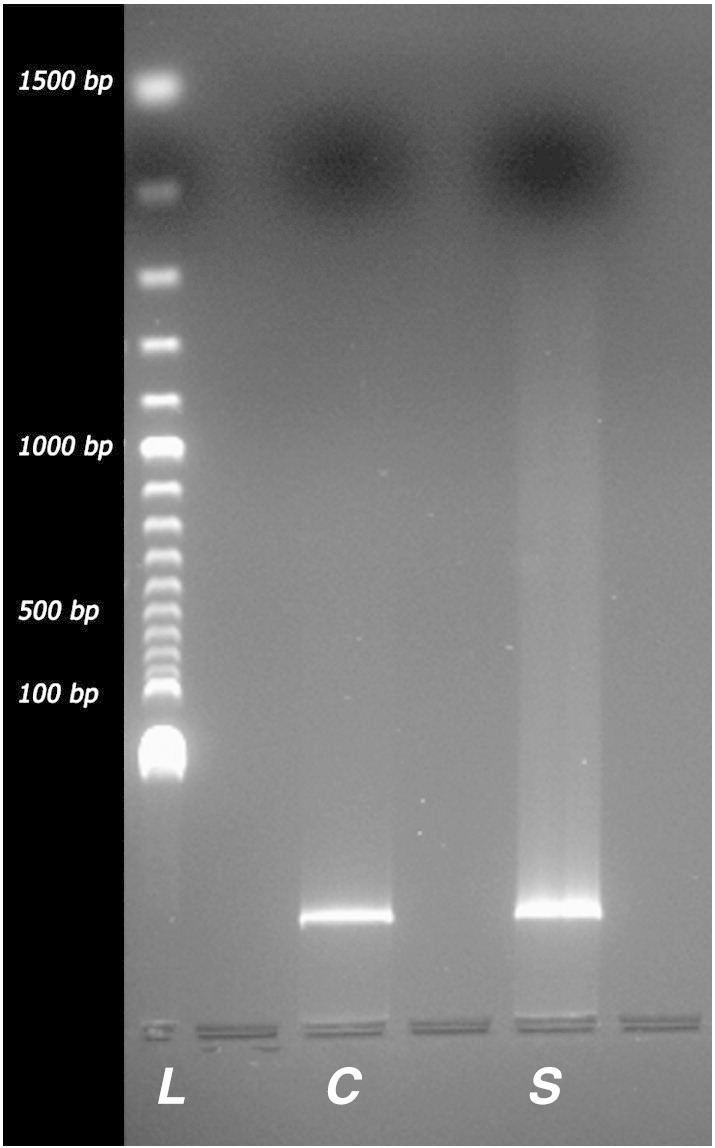
DNA integrity gel analysis. L. DNA ladder at 100 bp intervals. C. Control unwounded tendon (n = 6). S. Suture wounded tendon (n = 6). Note; gel shows a nuclear smear and no evidence of 180 bp laddering associated with apoptosis.

Control tendons placed in live/dead solution containing ethidium homodimer and calcien AM without tension were found to maintain a predominantly green cell fluorescence indicating these cells remained viable for the 12 h duration (data not shown). Unwounded tendon placed under tension showed some cell death at the anchored ends of the tendon but in the centre of the tendon explants, the cells remained green for the duration of the time lapse capture. Tendons sutured with a 50% grasping suture with applied tension demonstrated an area of acellularity (Fig. 1) and there was an area of progressive cell death immediately around the suture. Tendons sutured with a 50% grasping suture, not under tension, showed some dead cells around the suture grasp but no formation of an acellular zone. Cell death was significantly greater in tendon sutured under tension (*p* < 0.05). DNA integrity analysis showed a typical DNA smear suggesting that there had been cell necrosis, whereas laddering associated with the process of apoptosis was absent (Fig. 2).

### 2.2 *In vivo* cell biology of the fate of the acellular zone

#### 2.2.1 Formation and persistence of the acellular zone

**Fig. 3:**
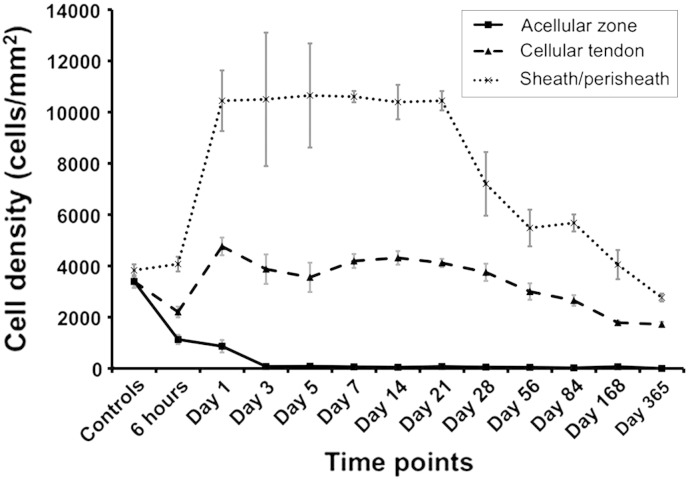
Temporal cellular density changes following suture insertion**.** Cells were quantified in the acellular zone, cellular tendon, and sheath/perisheath (a.k.a. subcutaneous tissue) in order to calculate the mean number of cells per mm^2^ at different time points. Within 6 h of suture there were significant changes in the cellularity of the sheath and the tendon that persisted for 1 year (p = 0.039) indicated by an asterisk. Error bars denote standard error of mean.

Following suturing tendon, the acellular zone formed rapidly and persisted for one year with no evidence of cellular repopulation of the matrix. Six hours after suturing of tendon, cells were present within the area of the tendon grasped by suture. Evidence of an acellular zone formation could be seen at 6 h but the acellular zone was not fully defined until 24 h following injury. The surrounding tissues became increasingly cellular and with peak cellularity at 24 h and persisted for 21 days. Tendon cellularity initially dropped at 6 h following suture insertion, and then the cellularity of the tendon remained elevated for up to 84 days (Fig. 3).

#### 2.2.2 Ultrastructural changes of the cells in the acellular zone following suture

**Fig. 4:**
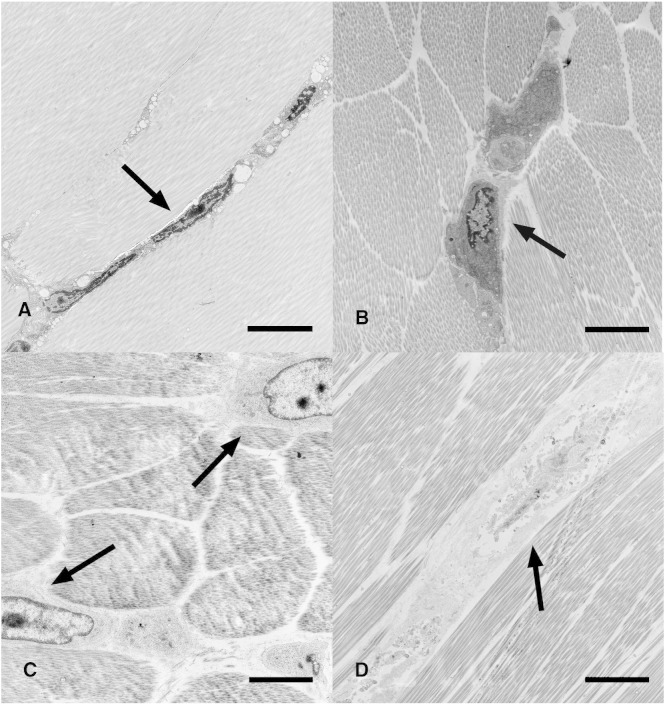
Electron micrographs of tenocytes in situ A. Tenocytes in unwounded flexor tendon show thin elongated nuclei and cytoplasmic membranes in contact with (arrow) neighbouring cells. B. Tenocytes fixed immediately following grasping by suture. Cells are rounder in cross-section (arrow) than those in unwounded tendon and cell contacts less obvious. C. tenocytes 3 h following grasping suture. Cell processes are lost between cells and chromatin condensation can be seen in nucleus (arrows). D. Tenocytes 24 h after suture grasp. Fragmentation of nuclei, breakdown of cells and absence of plasma membrane (arrow) is most evident. Scale bar represents 5 μm.

Cell morphology in unsutured tendon showed long thin elongated nuclei that have long cytoplasmic processes, which allowed contact with neighboring cells (Fig. 4-A). Following insertion of the suture, the cells became swollen and rounder in morphology (Fig. 4-B). Clumps of chromatin could be seen in the nuclei and the cells separated as the cytoplasm swelled (Fig. 4-C). After 24 h there was loss of plasma membrane integrity and nuclear fragmentation as the cells necrosed.

#### 2.2.3 Apoptosis of surrounding cells

**Fig. 5:**
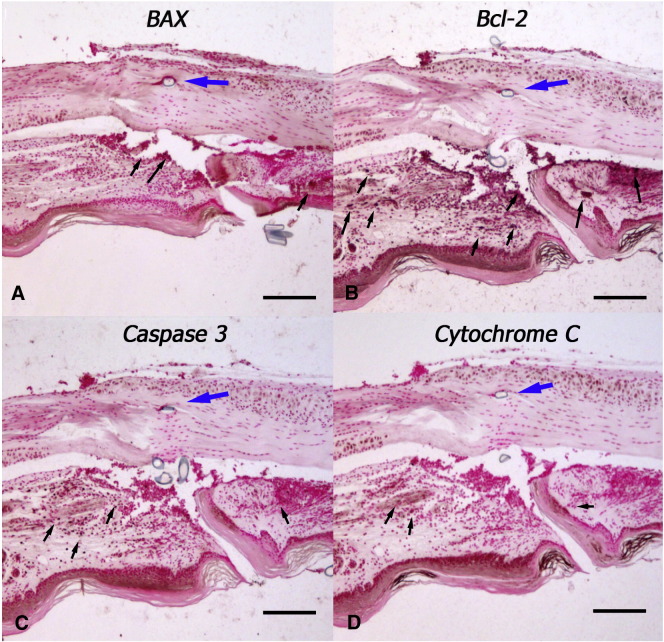
Cellular staining for apoptotic markers in sutured tendon harvested at 6 h post injury. A. Bax, B. Bcl 2, C. Caspase 3, D. Cytochrome C. Suture is indicated by the blue arrow. Small black arrows indicate areas of antibody expression. Scale bar represents 200 μm.

Evidence of apoptotic markers, Bax, Bcl-2, caspase 3, and cytochrome C was not observed in the tendon 6 h following suture or at a subsequent seven day and six month time points, however expression of these markers was evident in the subcutaneous tissues (ST) (Fig. 5).

#### 2.2.4 Disruption of cytoskeleton with cells under tensional load

**Fig. 6:**
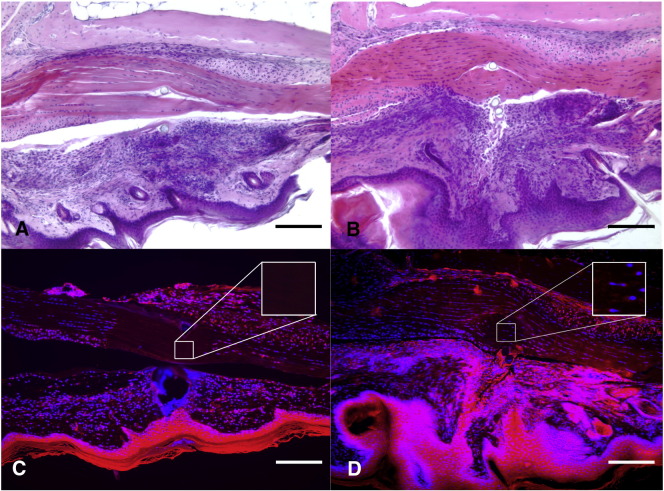
Representative images of tendon 24 h after suture *in vivo***.** Haematoxylin and Eosin staining (A and B) and Hoescht (Blue) and TRITC phalloidin (red) (C and D). A. Mouse FDP tendon sutured under standard physiological tension with acellular zone formation. B. Tendon sutured with tension abolished by proximal tenotomy with no acellular zone formation. C. Loss of actin staining in the acellular zone following grasping suture under tension. D. Actin staining and no acellular zone formation in tendon with tension abolished through proximal tenotomy. Scale bar represents 200 μm.

TRITC-Phalloidin staining for F actin was not evident in the acellular zone at any of the time points, indicating that disruption of the actin cytoskeleton network had occurred in the tendon cells that had been sutured under tension (Fig. 6-C). The abolition of tension through proximal tenotomy prevented the acellular zone from forming and cells appeared to maintain close proximity in rows (Fig. 6-D).

#### 2.2.5 Persistence of inflammation following suture

**Fig. 7:**
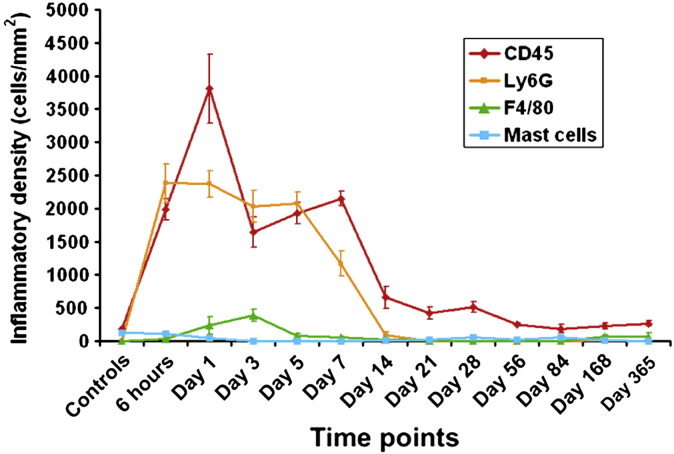
Temporal inflammatory cell activity changes following suture insertion. Cell labeling of CD45, ly6G, F4/80 and mast cells was performed. There is an increase in CD45 and Ly6G+ve cells in the subcutaneous tissues that persist for 14 days. Error bars denote standard error of mean.

**Fig. 8:**
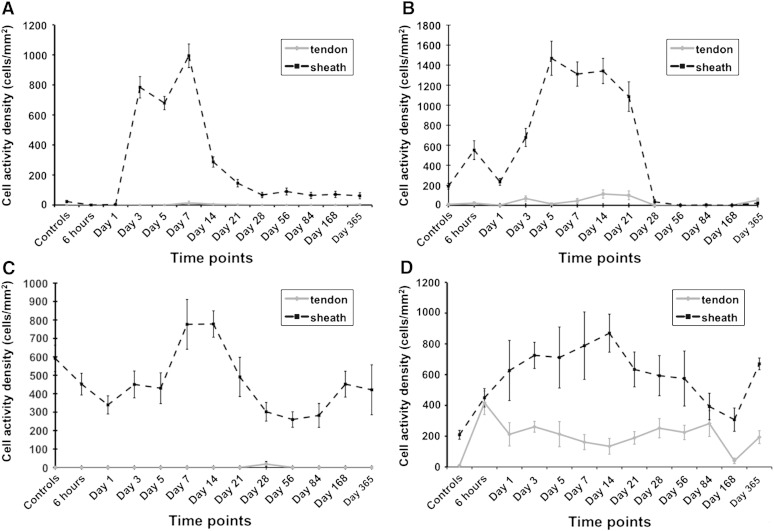
Temporal cellular activity changes following suture insertion**.** The grey lined graph represents changes in the tendon and the dotted lined graph represents changes in subcutaneous tissues (ST). A. Cell proliferation (BrdU). B. Type 1 collagen synthesis (Hsp 47). C. Vascularisation and myofibroblast expression (α-SMA). D. Apoptosis/necrosis (TUNEL). Changes in activity of BrdU, Hsp 47 and α-SMA occur mainly in the ST, whereas apoptosis occurs in both the tendon and ST. Error bars denote standard error of mean.

**Fig. 9:**
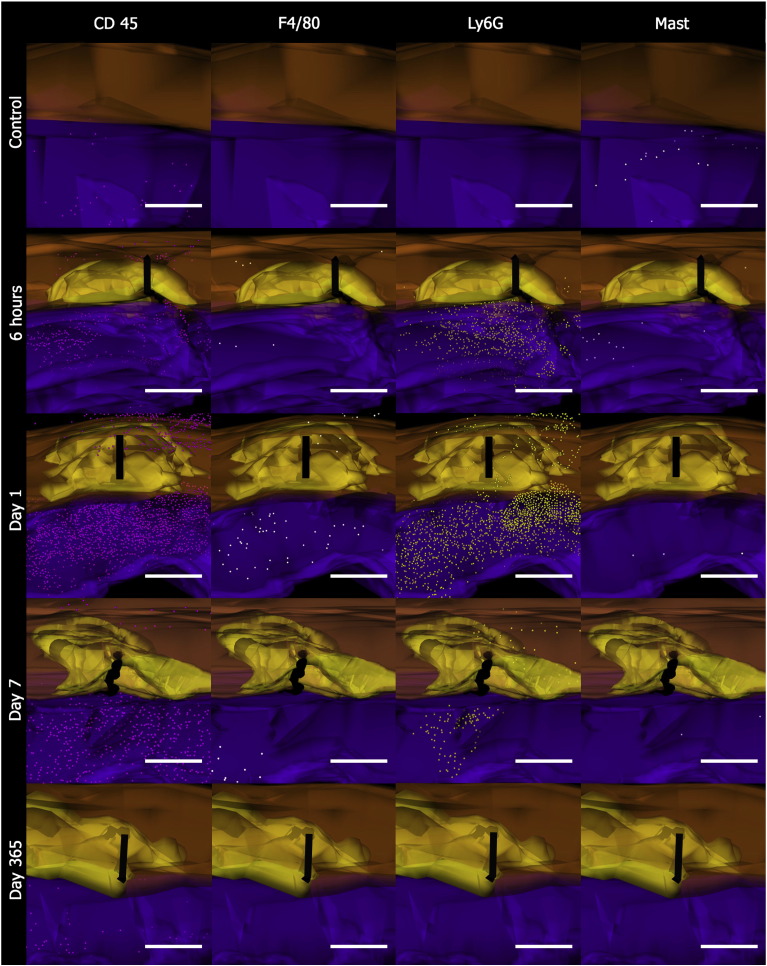
3D cellular mapping of inflammatory cell activity. Images of controls, 6 h, Day 1, Day 7, and Day 365 after suture. CD45+ve cells (pink), F480+ve cells (white), Ly6G+ve cells (yellow), Toludine blue+ve mast cells (silver). Tendon is represented in orange and subcutaneous tissue (ST) is represented in purple. The grasping suture is represented in black, and the acellular zone represented in yellow. Scale bar represents 200 μm.

**Fig. 10:**
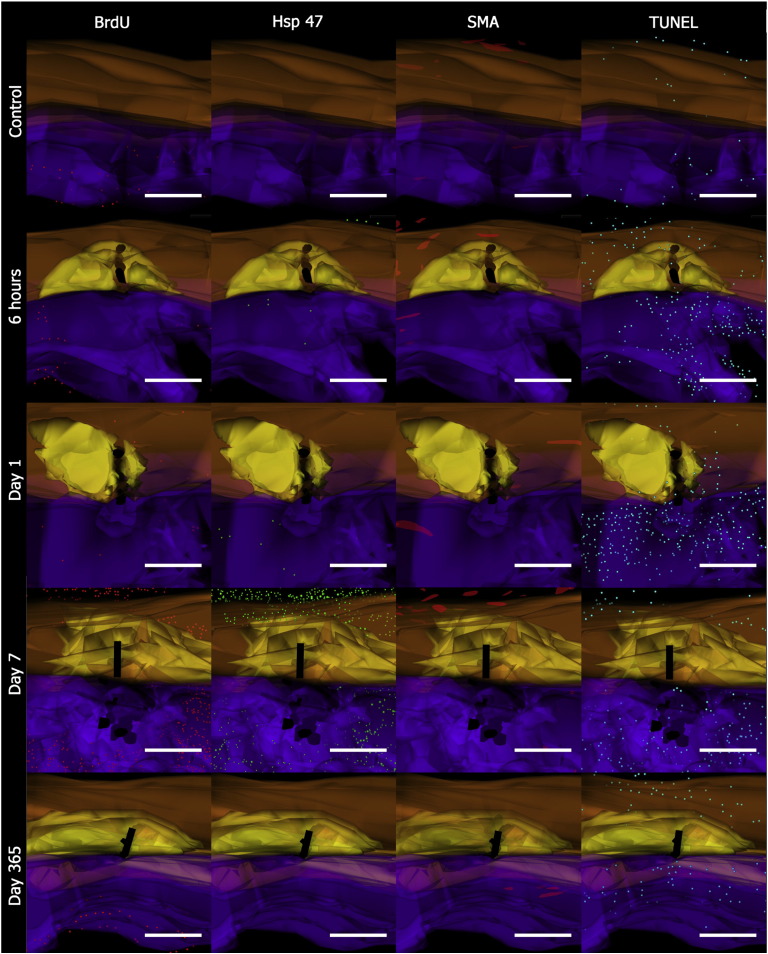
3D cellular mapping of cell activity**.** Image of controls, 6 h, Day 1, Day 7, and Day 365 after suture. BrdU+ve cells (red), Hsp 47+ve cells (green), α-SMA+vessels (red), TUNEL+ve cells (blue). Tendon is represented in orange and subcutaneous tissue (ST) is represented in purple. The grasping suture is represented in black, and the acellular zone represented in yellow. Scale bar represents 200 μm.

In the early phases of healing, CD45 and Ly6G were typically elevated in response to tissue injury (Fig. 7) but few inflammatory cells were seen to infiltrate the tendon tissue. The inflammatory cell reaction persisted for 14 days at a significantly elevated level compared with controls (*p* < 0.05). Staining for markers of proliferation, synthesis, myofibroblast/pericytes, apoptosis and inflammation showed that most of the cellular activity occurred around the suture in the surrounding subcutaneous tissue (ST) (Fig. 8). 3D cellular mapping demonstrated that the vast majority of CD45 expressing cells co-expressed for neutrophil marker, Ly6G, at the early time points. CD45 expression persisted diffusely in the ST whereas Ly6G expression appeared to be localised around the suture forming a cuff of cells (Fig. 9). Mast cells stained with toludine blue were present in the dermis at 6 h post wounding but were then reduced in number for 28 days. Expression of F4/80 was generally low with peak expression occurring at day 3 and confined to the ST. Hsp 47 and BrdU staining also increased at day 3 in fibroblasts around the zone of injury. This expression was mainly confined to the ST (Fig. 10) and around the tissues situated dorsally and apposed to bone i.e. the vinculum. Peak levels of Hsp47 and BrdU labeling occurred between days 7 and 14. The epidermis was a notable site for BrdU staining and TUNEL staining. Peak staining of TUNEL was seen at day 7 mainly confined to the subcutaneous tissues and some areas of the tendon. There was a gradual decline in BrdU and TUNEL staining after 21 days and HSP 47 activity in the tissues dropped to baseline levels after 28 days. The expression of α-SMA declined slightly after cutting the subcutaneous tissues but then showed increasing expression by days 7 to 14 that slowly reduced to baseline levels over the course of the year (Fig. 8C).

**Fig. 11:**
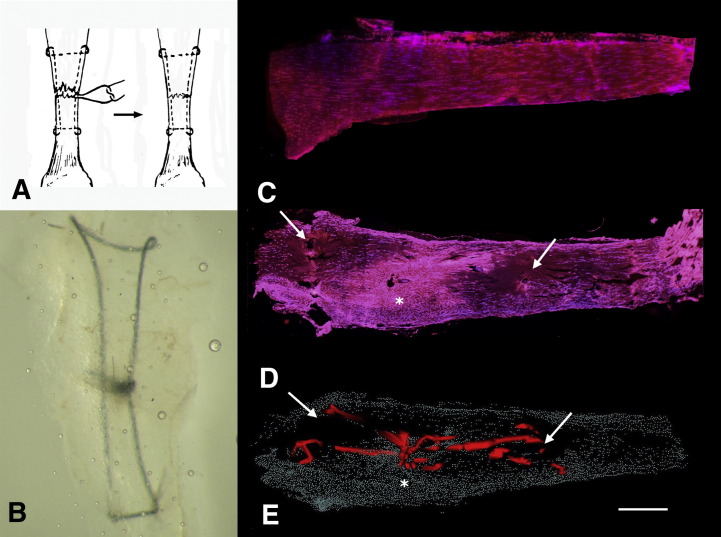
Distribution of acellular zones following a modified Kessler Repair in Achilles tendon (stained with Hoechst 33258 and TRITC Phalloidin). (A) Schematic of modified Kessler repair being performed (B) Modified Kessler repair performed in mouse Achilles tendon cleared in glycerol (C) Normal unwounded mouse Achilles tendon demonstrating well defined linear rows of cells. (D) Kessler sutured mouse Achilles tendon at 3 weeks post wounding demonstrating hypercellularity at point of partial laceration and suture knot (asterisk) and large areas of acellularity related to the grasping components of the Kessler (white arrows). (E) 3D reconstruction of Kessler suture (red) in Achilles tendon three weeks following wounding. Note large acellular zones in high strain areas of the grasping components of the Kessler suture (white arrow). Scale bar = 500 μm. (For interpretation of the references to colour in this figure legend, the reader is referred to the web of this article.)

### 2.3 Kessler repair model

Acellular zones could be seen to form in the grasping regions of the modified Kessler repair. Three dimensional reconstructions of this shows the acellularity was more extensive forming at all four grasping corners of the Kessler repair and along the axis of the core sutures (Fig. 7). Notably the area of tendon under greatest strain was outwith the suture repair, distally and proximally, highlighted by the acellularity of the matrix, whereas the tendon within the boundaries of the repair were more cellular and presumably under less strain.

## 3 Discussion

The primary suture repair of tendon can be dated back to Galen who later condemned the practice in his scriptures, *Ars Parva* (Manske, 2004). Subsequent surgery on tendon was revived by the work of Arab surgeons Rhazes, Avicenna and Abulcasis in tenth century A.D. (Kleinert, Spokevicius et al. 1995). Today, the primary repair of divided tendon ends in the acute clinical setting is common practice but it is unclear what effects suturing has on the cell biology of tendon. Our study showed that the grasping component of the suture forms acellular zones in flexor and Achilles tendons, whether this relates to a simple grasping suture or modified Kessler repair. This is a phenomenon that occurs in both intrasynovial and extrasynovial tendon hence is likely to arise in many clinical situations. Modifications of the Kessler repair are currently the most utilised repair method in the Western world (Tang, 2006) and performed on both hand flexor tendon lacerations and Achilles tendon ruptures. The validation of many tendon repair surgical techniques currently used in clinical practice is performed in cadaveric tissue but some studies have suggested tendon weakens after suture repair in living tissue (McDowell, Marqueen et al., 2002). Silva et al. had shown in canine flexor tendon insertion site injuries that the suturing of tendon resulted in significant softening of the tendon ends, resulting in a 45% drop in tensile strength at 10 days and 21 days following suture (Silva, Boyer et al., 2002). This highlights the importance of conducting studies on suture methodology on *in vivo* models, whereby the effect of inflammation and local cell damage can be considered.

The present study has shown that a zone of acellularity begins to form within hours of suturing the tendon and that tension through the tendon is required for its formation. Furthermore due to mechanical traction, the tenocytes move out of the zone of grasp as their intercellular cytoplasmic contacts appeared to be disrupted. Studies have shown that tenocytes slide in rows, between tendon fascicles (Screen, Lee et al. 2003). The loss of cell-to-cell contacts through mechanical forces suggests cells are retracted away from the zone of injury, as opposed to migrating. Cell retraction is associated with rapid F actin reorganization, mediated by Rho kinase (Groeger and Nobes, 2007), and dephosphorylation of focal adhesion proteins (O'Brien, Kinch et al. 1997). The rapid cell retraction of tendon fibroblasts following suture may limit the degree of damage to the neighboring cells. McNeilly et al. previously had shown that the cells connect through 3D cellular networks linked together by gap junctions that were likely to communicate mechanical loads and stresses to neighboring cells (McNeilly, Banes et al. 1996). Preservation of these cell-to-cell contacts would seem important for normal tendon homeostasis. Numerous studies have shown that connexin 43 and gap junctions have an important role in cell signaling between cells in the tendon matrix (McNeilly et al., 1996, Ralphs et al., 2002). In rat glioma cell populations these hemichannels in conjunction with their gap junction counterparts have been shown to propagate cyto c mediated cell death to neighboring cells (Decrock, De Vuyst et al. 2009). There is notable cell death associated with suturing a tendon but there was little to suggest that this was due to recognized apoptotic pathways such as those related to Bax, Caspase 3 and Cytochrome C. Our results indicate that the local cell death was characteristic of necrosis in the absence of the classic laddering associated with apoptosis (Tilly and Hsueh, 1993). Previously we have shown that this segment of the tendon in the mouse is avascular (Wong, Bennett et al. 2006) which would tend to indicate that ischemia appears unlikely to contribute to acellular zone formation.

The ultrastructural changes including loss of cell-to-cell contacts, cytoplasmic swelling, chromatin condensation in the nuclei, followed by loss of plasma membrane integrity and nuclear disruption, occurred rapidly and are hallmarks of cellular necrosis (Krysko, Vanden Berghe et al. 2008). The rate at which the acellular zone forms in time lapse and *in vivo* studies in conjunction with tension suggests the cells move out of the zone of injury through cell retraction, in addition to cellular necrosis. Through the loss of cell-to-cell contacts immediately in the acellular zone, cells lose their tension. Loss of tension to tenocytes can have dramatic effects on the surrounding matrix. The unloading of tendon fascicles leads to a greater than 400 fold increase in gene expression of interstitial collagenase (MMP 13) and stromelysin (MMP 3) within 8 h which have a role in matrix degradation (Leigh, Abreu et al. 2008). This may lead to a microenvironment favorable for cell movement. Disruption to the cellular actin cytoskeleton may provide one mechanism for this to occur. Lavagnino et al. (2005) showed that *in vitro* disruption of tendon fibroblast actin cytoskeleton by cytochalasin D, resulted in increased catabolic interstitial collagenase activity and inhibition of α1(I) collagen mRNA expression. The same effect was accomplished by alleviating mechanical tension in tendon fibroblast gels which exhibited collagenase activity within 24 h until a new homeostatic level of tension was acquired at around 14 days (Lavagnino and Arnoczky, 2005). Once the tendon fibroblasts reestablished tension *in vitro* they began to express α1(I) collagen mRNA and stopped expressing interstitial collagenase. The observed mechanical tuning of cells to their microenvironment is supported by McDowell's (2002) observations that the mechanical strength of suture repaired tendons is markedly diminished for two weeks after repair (McDowell et al., 2002). These results suggest that the cells take around two weeks to establish an internal cytoskeleton tension through interactions with the local extracellular environment. Our studies demonstrated a persistence of the acellular zones up to a year following injury which gives rise to some concern over the clinical impact of leaving sutures *in situ*.

The inflammatory insult from the single suture is present for 14 days and then gradually decreases however cells do not appear to infiltrate the tendon substance. Little evidence of proliferation, synthesis, inflammation or apoptosis occurred in the tendon substance. These activities are far more evident in the surrounding sheath and ST, which followed a recognized pattern of wound healing. Using inflammatory markers we have shown that there was an early drop in the number of mast cells, which was due to injury induced degranulation seen at 6 h followed by a peak of CD45+ve and Ly6G+ve cells at 24 h following injury. This was followed by a peak in F4/80 expressing macrophages and elevated CD45 labeled cells at 3 days and persisting elevation in CD45 count but not F4/80+ve cells at day 7 in the ST. It is evident that inflammatory cell activity around the tendon persists to 14 days due to the presence of neutrophils. Archambault et al. (2002) have demonstrated in the flexicell system that rabbit fibroblasts in conjunction with inflammatory cytokine Il-1β significantly increased expression of MMP1 and MMP3 which was further increased by applying cyclical stretch forces (Archambault, Tsuzaki et al. 2002). The environment we have created *in vivo* by the application of a suture and mobilization equates to a prolonged inflammatory environment with tendon fibroblasts under cyclical strain. Although MMP expression was not investigated in this particular study, it would appear that the acellular zone represents a physical manifestation of this degenerative response. This prolonged inflammatory activity may contribute to weakening of the tendon over this time period. Evidence from studies in rheumatoid tenosynovitis has shown that inflammation of the tendon sheath produces raised levels of proinflammatory cytokines and proteolytic enzymes such as MMP 1, 2 and 13 that can cause tendons to weaken and spontaneously rupture (Jain, Nanchahal et al. 2001). Zymography studies have demonstrated that MMP1 is the predominant matrix enzyme in spontaneously ruptured supraspinatous tendon (Riley, Curry et al. 2002). The relationship between matrix metalloproteinases, inflammation and acellular zone formation merits investigation.

The prolonged inflammatory activity surrounding the suture is mitigated by apoptotic activity in the surrounding tissues. Previously we found a similar inverse relationship between inflammation and apoptosis during the tendon adhesion formation process although more protracted (Wong, Lui et al. 2009) and a number of studies have shown that the process of cell death forms and important part of inflammatory cell clearance in wound healing (Chowdhury et al., 2006, Poon et al., 2009).

Although the small animal numbers may be perceived as a limitation of the study, the experimentation was powered accordingly, based on our previous data that showed the *in vivo* suture model to be highly reproducible with low inter-animal variability. Our move towards using a combination of *ex vivo* and *in vivo* techniques were aimed to isolate and image the effect of suture and tension on tendon with and without the systemic influences of inflammation. We have shown that cell death plays a role at numerous points in the surgical suture of tissue and suturing of tendon has been shown in a number studies to induce biomechanical weakening of the tendon (Reynolds et al., 1976, Bishop et al., 1986, McDowell et al., 2002, Zobitz et al., 2000) when performed in living tendon. The mechanism by which acellular zones form seem crucial to the loss in mechanical integrity as inducing acellular tendon using chemical treatments such as high concentration of cellular detergents have been shown not to impair the mechanical integrity of rat tendons (Cartmell and Dunn, 2000). Indeed acellular tissue reconstructions have been shown to be of benefit in reconstructing tendon injuries in clinical practice (Lee, 2007). The biochemical combination of necrosis, acellular zone formation and prolonged inflammation following suture may all contribute to tendon softening and each of these will be examined independently for their contribution in future studies. Numerous tissues exhibit evidence of the detrimental effect of suturing, like corneal tissue, which becomes hazy and can clearly be seen to have acellular zones following suture (Matsuda, Yoshiki et al. 1999), meniscal repair with sutures form acellular zones, which over time lead to joint degeneration and arthritic changes (Hunziker and Stahli, 2008). Cumulatively there is evidence to suggest that suturing unwounded tendon induces catabolic changes in the tendon. Importantly the study highlights the importance of tendon mechanobiology in maintaining normal tissue homeostasis and how simple surgical procedures can disrupt this biology significantly.

## 4 Methods

### 4.1 Live/dead tendon imaging

Nine middle digits, flexor digitorum profundus (FDP) tendons were harvested from freshly euthanized 10–12 week old C57/BLJ mice. Using an operating microscope (Leica MZ7.5, Germany) at 10–40 times magnification, the FDP tendon was dissected free. This was performed by making a longitudinal cut down the palmar surface of the digit from the tip of the digit to the palm. The pulleys and flexor digitorum superficialis (FDS) were sharply divided and FDP was divided at the level of the palm and at the distal insertion. Tendons harvested measured approximately 1 cm in length. Harvested tendons were immediately placed in sterile CO_2_ independent media (Gibco, Ayrshire, UK) and Live/Dead assay kit solution (Molecular Probes, Oregon, USA) which contained 2 μM calcein AM and 4 μM EthD-1. Tendons bathed in this solution were incubated at 37 °C, 5% CO_2_ for 1 h prior to inspection by confocal microscopy.

Calcein AM dye enters living cells and ethidium homodimer (EthD-1) is taken up by dead cells. Calcein AM fluoresces green when activated by intracellular esterase activity in living cells (excitation 495 nm and emission 515 nm). EthD-1 enters damaged cell membranes and fluoresces red on binding to nucleic acids (excitation 495 nm and emission 635 nm) but is excluded by living cell membranes.

Three experimental groups were performed in triplicate; control uninjured tensioned tendon, sutured tensioned tendon, and sutured untensioned tendon. Experiments were performed on 35 mm cover glass bottom sterile culture dishes (Fluorodish, World Precision Instruments Inc, CA, U.S.A.) with a thin layer of Sylgard (184 silicone Elastomer, Dow chemical, MI, U.S.A.) placed along the outer rim of the well. Sylgard lined culture dishes were incubated overnight at 55 °C to induce polymerization. Fixed position posts were anchored into the Sylgard rim in each dish using 0.1 mm diameter insect pins (Fine Science Tools GMbd, Germany).

Control tensioned tendons were sutured at both ends with 10/0 polyamide sutures (BBraun Medical, Sheffield, U.K.). The sutures at the ends of the tendon were tensioned around the insect pins anchored into the Sylgard until the tendon was taught. The ends of the tendon were at least 3 mm away from the area to be imaged. The procedure was repeated for the sutured tensioned tendon. This was kept taught as above but a further 10/0 single grasping suture, grasping approximately 50% of the tendon fibres (Wong, Cerovac, Ferguson and McGrouther, 2006), was placed in the middle of the tendon in the region of image capture.

Sutured untensioned tendon was sutured as above in the middle of the tendon with a 10/0 single grasping suture. After placement of the single grasping suture, tension was immediately released from the tendon by removing the insect pins. Subsequently the samples were bathed in Live Dead solution and images were captured using a Biorad MRC 1024 Multiphoton confocal microscope (Biorad, CA, U.S.A.) with Spectra Physics Tsunami Infrared Laser (750 nm) (Newport Solutions, CA, U.S.A). Images were captured at two hourly intervals at 20× magnification and 2 μm thickness z stack slices for 12 h.

### 4.2 Analysis of DNA integrity

Six mice were used to analyse DNA integrity following suture using the standardised single suture injury model. The mouse tendon was harvested 24 h post injury and snap frozen in liquid nitrogen. Uninjured tendon was harvested from the contralateral limb of each mouse and acted as the controls. Six biological replicates were completed and pooled into wounded and unwounded sample groups for comparison. The DNA extraction protocol was adapted from that described by Tilly and Hsueh (1993). Pooled tendon was homogenised by pestle and mortar in liquid nitrogen. Further homogenisation was obtained by placing samples in 100 mM NaCl, 10 mM EDTA, 300 mM Tris HCl with a pH of 8.0; 200 mM sucrose, and 0.65% SDS before incubation for 30 min at 60 °C. The sample was then incubated for 1 h at 55 °C after Proteinase K was added at 500 μg/ml. Following the addition of Potassium acetate at 500 μg/ml, the sample was placed on ice for 30 min. Following centrifugation at 4500 rpm for 15 min at 4 °C, the supernatant was extracted once with phenol:chloroform:isoamylalcohol (25:24:1, by vol.). This mixture was further processed by the addition of chloroform:isoamylalcohol (24:1, v/v), and the DNA was precipitated with absolute ethanol. The DNA was dissolved in 200 μl TE, pH 8.0, and incubated with 2.5 μg DNase–free RNase (Sigma-Aldrich, USA) for 60 min at 37 °C. Following further phenol extraction and phenol precipitation, equal amounts of DNA were resolved on 2% agarose gels.

### 4.3 Transmission electron microscopy

Samples were prepared for electron microscopy as described previously (Canty, Lu et al. 2004). Briefly, mouse digital flexor tendons were sutured *in vivo* and harvested immediately, at 3 h post suture and at 24 h post suture, and were immediately fixed in 2% glutaraldehyde in 100 mM phosphate buffer, pH 7.0, for 30 min at room temperature. Control mouse digital flexor tendons were also harvested in an identical fashion from unwounded mice. Samples were then post fixed for 2 h at 4 °C in fresh fixative. After washing in 200 mM phosphate buffer, the samples were fixed in 1% glutaraldehyde and 1% OsO_4_ in 50 mM phosphate buffer, pH 6.2, for 40 min at 4 °C. After being rinsed in distilled water the samples were stained en bloc with 1% aqueous uranyl acetate for 16 h at 4 °C, dehydrated and embedded in Spurrs’ resin. Ultra-thin sections, ∼ 60 nm thick, were cut for normal transmission electron microscopy and were collected on uncoated copper 200 grids. Serial sections were taken and placed on formvar-coated copper 1000 μm slot grids, stabilized with carbon film. All sections were subsequently stained with uranyl acetate lead citrate, and examined using a Philips BioTwin transmission electron microscope (Phillips Electron Optics, The Netherlands). Images were recorded on 4489 film (Kodak, UK) and scanned using an Imacon Flextight 848 scanner (Hasselblad, USA).

### 4.4 *In vivo* studies

All animal procedures were approved by the Local Ethical Committee at the University of Manchester and complied with British Home Office regulations on the care and use of laboratory animals. Our limitations in sample size per time point were justified from our previous studies where we were able to show that the variability between sutured animals, taking into account suture technique and reproducibility was small. Appropriate power calculations were used to calculate sample size.

39 mice were used for immunohistochemical studies and 3D reconstruction with cellular mapping. Our previously described model was used: this involved a standardised procedure; which used a single interrupted suture of a digital flexor tendon (Wong et al., 2006).

The *in vivo* study used the hind paw deep digital flexor of male C57/BL6 mice aged between 10 and 12 weeks (25–30 g). Surgery was performed under a standard mouse general anaesthetic protocol (Induction using 4% isofluorane (Abbott, UK) and 4 l/min oxygen driver, maintenance 2% isofluorane with 2 l/min oxygen driver and 1.5 l/min nitrous oxide). The left hindlimb was cleaned with 70% ethanol and blood flow to the operative field was reduced through application of a tourniquet using dressing elastic to the popliteal fossa. The anaesthetised mouse was positioned under a Leica MZ7 operating microscope (Leica Microsystems, Germany) and operated upon at 10 to 40 times magnification.

Second, third and fourth digits were suture wounded to study stress fibre architecture, cellular function markers and inflammatory markers respectively. Six control, unsutured tendons were taken from several corresponding digits on the contralateral paw in the corresponding area of injury.

A skin incision was made at the midpoint of the middle phalanx of each digit of the left hind paw to expose the tendon. The wound was regularly irrigated with 0.9% saline to avoid tendon dehydration.

A single 10-0 polyamide suture (BBraun Medical, UK) was inserted transversely through approximately 50% of the deep digital flexor tendon diameter and tied.

Following the application of the tendon suture, skin wounds were closed with a single 10-0 polyamide suture, the tourniquet removed and pressure applied to the wound to ensure haemostasis. The hind paws were cleaned with 0.9% saline and the wounds were left exposed to dry. The mice were allowed to mobilise freely and three mice were euthanized at 6 h, then at 1, 3, 5, 7, 14, 21, 28, 56 and 84, 168 and 365 days. Three mice had a slight variation in the operative protocol and underwent the same exposure and tendon wounding but had a further wound made at the level of the tarsal bones to allow for a proximal tenotomy of the deep digital flexor tendon. Hind paws were flexed and extended to ensure that normal tenodesis was obliterated hence removing traction forces from the sutured tendon site. This group of mice were euthanized 24 h following suturing and their tendons collected.

Four hours prior to mice euthanasia each mouse was injected with Bromodeoxyuridine (5-bromo-2-deoxyuridine, BrdU) injected into the peritoneal cavity at a dose of 10 μl/g of mouse.

The hind limbs were immersed in zinc fixative (Beckstead, 1994) for 48 h at 4 °C. The third digit of each sample was excised and decalcified in 20% EDTA for 15 days, with solution changes every 5 days, and then embedded in paraffin wax. The fourth digit soft tissue was filleted off the bone and stored in 70% ethanol until they were ready to be embedded in paraffin wax. Serial sections, 7 μm thick, were cut from paraffin embedded blocks. Four sections were mounted onto 4% APES and 1% Poly-l-lysine coated slides, and were dried at 37 °C for 24 h.

The central slide was identified from the sections by staining alternate slides for Harris haematoxylin (Raymond A Lamb, U.K.) and eosin (Raymond A Lamb, U.K.) or Hoechst 33258 bisbenzimidazole dye (Sigma Chemical Co., St Louis, USA) and TRITC labeled Phalloidin (Sigma-Aldrich, St Louis, USA) at the concentration of 1: 1000 and 1:500 overnight at 4 °C respectively. For the third digit, three sections from selected from around the central slide were antibody labelled (Supplement 1) for BrdU (marker for proliferation), Heat shock protein 47 (Hsp 47 or “collingin”, marker for type 1 collagen synthesis), alpha smooth muscle actin, (α-SMA, marker for pericytes and myofibroblasts) and TUNEL (marker for DNA fragmentation/apoptosis). The fourth digit was serially sectioned and antibody stained with CD45 (pan leucocyte marker), Ly6G (neutrophil marker), F4/80 (activated macrophage marker), and toludine blue (mast cell stain) in triplicate per mouse digit. Labeling for apoptotic markers Bax (apoptotic marker), Bcl-2 (anti-apoptotic marker), Caspase 3 (apoptotic marker) and Cytochrome C (apoptotic marker) were performed at time points 6 h, 7 days and 6 months to look for evidence of apoptotic cells. Immunoperoxidase techniques were standardised following initial dilution studies to establish optimal dilutions and conditions for all primary antibodies. For mouse monoclonal antibodies a specific mouse on mouse (MOM) kit (Vector Laboratories) was used. For rat monoclonal antibodies, a standard rabbit anti rat biotinylated secondary antibody was used and amplified using the elite ABC kit (Vector laboratories). For rabbit polyclonal antibodies the rabbit ImmPRESS biotinylated kit was used. These kits were used as recommended in the manufacturer's guidelines. The TUNEL kit (Roche) was used with peroxidase conversion which allowed for assessment by 3,3′ diaminobenzidine (DAB) substrate staining. Antibody labeling of samples was undertaken using the protocol described below (Table 1). Samples were washed twice for 5 min using 0.1% tween (v/v) in phosphate buffered solution (PBS) between each step of the protocol.

**Table 1:** Antibodies, pretreatments, reagents, and controls used for labeling samples. Incubation performed at room temp (RT) or 37 °C. ABC represents avidin–biotin complex.

Antibody	Pretreatment	Blocking solution	Primary incubation	Secondary incubation	ABC	Substrate	Control tissue
BrdU (Abcam)	10 min in 4 M HCL, 5 min in borate buffer	1% rabbit serum for 1 h at room temp (RT)	1:200 for 1 h 37 °C	1:200 rabbit anti-rat biotinylated IgG for 15 min RT	Yes	DAB	Spleen
Hsp-47 (Stressgen)	None	MOM block for 1 h RT	1:200 for 1 h 37 °C	MOM kit 2 IgG for 10 min RT	Yes	DAB	Skin wounds
α-SMA (Abcam)	None	2.5% goat serum for 1 h RT	1:200 for 1 h 37 °C	ImmPRESS kit for 30 min RT	No	DAB	Spleen
TUNEL (Roche)	30 min in Tris HCL +	None	2:3 for 1 h 37 °C	1:2:1 Sheep serum:PBS: POD kit for 30 min RT	No	DAB	Large intestine
CD45 (BD pharmingen)	None	1% rabbit serum for 1 h RT	1:100 for 1 h 37 °C	1:200 rabbit anti-rat biotinylated IgG for 30 min RT	Yes	DAB	Spleen
F4/80 (Serotec)	None	1% rabbit serum for 1 h RT	1:200 for 1 h 37 °C	1:200 rabbit anti-rat biotinylated IgG for 30 min RT	Yes	DAB	Spleen
Ly6G (BD pharmingen)	None	1% rabbit serum for 1 h RT	1:200 for 1 h 37 °C	1:200 rabbit anti-rat biotinylated IgG for 30 min RT	Yes	DAB	Skin wounds
Bcl-2 (BD pharmingen)	None	2.5% goat serum for 1 h RT	1:400 for 1 h 37 °C	ImmPRESS kit for 30 min RT	No	DAB	Skin and intestine
Bax (BD pharmingen)	None	2.5% goat serum for 1 h RT	1:400 for 1 h 37 °C	ImmPRESS kit for 30 min RT	No	DAB	Skin and intestine
Caspase 3 (R & D System)	None	2.5% goat serum for 1 h RT	1:200 for 1 h 37 °C	ImmPRESS kit for 30 min RT	No	DAB	Skin and intestine
Cytochrome C (Abcam)	None	2.5% goat serum for 1 h RT	1:200 for 1 h 37 °C	ImmPRESS kit for 30 min RT	No	DAB	Brain

Images of all stained sections of tendon with suture were obtained using a Leica DMRB Microscope (Leica Microsystems, Germany) and a Spot camera (Diagnostic Instruments Inc, USA) onto a silicon graphics PC (SGI, UK) and saved using Spot advanced software (Diagnostic instruments inc, U.S.A.) as .tiff files.

### 4.5 Calculating cellular changes in tendon, sheath and skin

Acellular zone variables were acquired as described from previous work on three sections that were 28 μm apart (Wong et al., 2006). Cells were counted in the acellular zone, the cellular tendon, and the sheath/subcutaneous tissues (ST) in specified sampling areas. The cell densities for any given area could therefore be calculated as cells/mm^2^. Briefly, from the selected sections the acellular zone outline was defined by joining the peripheral nuclei at its margins. The area was measured by image analysis software (Image Pro Plus version 4.5, Media Cybernetics, USA).

The tissue sample area consisted of the area of tendon with the suture in the centre of the image captured. To calculate the cellularity of samples, Image Pro Plus software was calibrated to colour match the nuclei on the slides and individual nuclei were counted within defined fields of view (Supplement 2). Six fields of view were selected for calculating cellularity and cellular expression of antibodies. Cell counts were performed on three fields of view for the tendon (Supplement 2-A, B, and C) and three fields of view for the sheath and subcutaneous tissues (ST) on three different sections (Supplement 2-D, E, and F). The total number of fields analysed per digit was 18 fields (nine tendon and nine sheath/subcutaneous tissue fields). Each field measured 50 μm by 200 μm except the acellular zone field that was measured according to the area of acellularity formed (Supplement 2-A). Cells were counted using the Image Pro Plus software. The mean cell density was calculated from the three fields in their respective tissues (tendon and sheath/subcutaneous tissues). This sampling method was also used for quantification of immunohistochemical markers. For the inflammatory profile where inflammatory cells were not seen in the tendon substance, a mean cell density of expression was taken from three fields of view surrounding the sheath, proximal, adjacent and distal to the area of injury.

### 4.6 Three dimensional reconstruction and cellular mapping

Three dimensional reconstruction of electron microscopic topography using Reconstruct (Fiala, 2005) has previously been described. We have applied the same methodology to serial sectioned and aligned immunohistochemically labeled samples. Briefly, serial sectioned histology and immunohistochemical image files were calibrated and imported as .tiff files into the Reconstruct program. Wire maps were produced by tracing individual structures such as tendon, blood vessels and the subcutaneous tissues. The acellular zone was also mapped by tracing around the zone for ease of identification on the reconstructed images. Distances between each image were calibrated and individual cells were mapped according to their stereological position and reproduced using the program's sphere option. Boissonnat surface shading and colour selection and transparency features were introduced after processing of the three dimensional reconstruction. Tendon was highlighted in orange, the acellular zone was yellow, the subcutaneous tissue was purple, and the suture was black. CD45 positive (+ ve) cells were labeled in pink, F4/80+ve cells in white, Ly6G+ve cells in yellow, and mast cells in silver. BrdU expression was mapped in red, Hsp47+ve cells were mapped in green, α-SMA+ve vessels were mapped in red, and TUNEL expression was mapped in blue.

### 4.7 Kessler repair model

Six C57 BL6 mice had modified Kessler repairs performed on their left Achilles tendon. Briefly, under standard anaesthetic protocol, hair bearing areas over the Achilles tendon were shaved, a tourniquet was applied to the mouse left hindpaw lower limb, and a longitudinal skin was made along the dorsal aspect over the distal mouse tibia. The Achilles tendon was exposed and a 50% division of the tendon was performed using microscissors. Using a 10/0 polyamide suture, a modified Kessler repair was performed (Kessler and Nissim, 1969). Mice were allowed to mobilise freely and were harvested 3 weeks following injury. Unwounded Achilles tendons were also harvested as controls. The tendons were carefully dissected from the hindlimbs and fixed in zinc fixative for 48 h at 4 °C. Tendon was processed and paraffin wax embedded for serial sectioning as above. Sections were cut at 7 μm thickness, then mounted on onto 4% APES and 1% Poly-l-lysine coated slides, dried for 24 h at 37 °C prior to staining with Hoechst 33258 and TRITC Phalloidin staining for 3D reconstruction. Images were captured with a standard fluorescent microscope (Leica DM RB light microscope, Switzerland).

### 4.8 Statistics

Data were analysed for normal distribution by plotting data on a histogram using SPSS 15.0 (SPSS Inc, Chicago, USA). Mean values were calculated using SPSS and expressed with the standard error of mean in brackets. The changes in cellularity seen at different time points were tested for significance using one way ANOVA and further analysed using a post hoc Tukey test. In all cases, the P value was considered significant if below 0.05.

## Figures and Tables

**Supplement 1:**
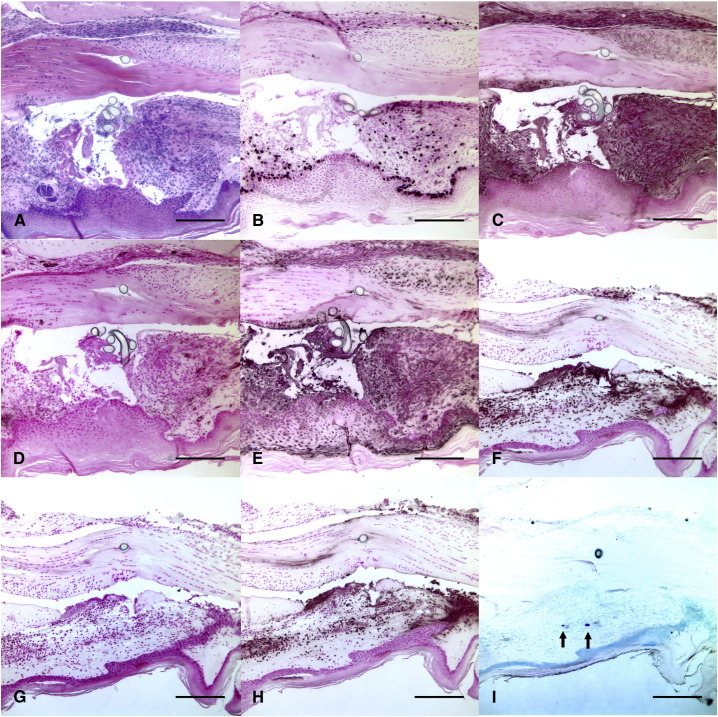
Representative sections from serial sectioned immunohistochemistry**.** Day 7 staining of sutured tendon A. Haematoxylin and Eosin staining, B. BrdU proliferation staining, C. Heat shock protein 47 expression, D alpha smooth muscle actin expression, E. TUNEL staining. Day 1 staining of sutured tendon. F. CD45 pan leukocyte staining, G. F4/80 activated macrophage staining, H. Ly6G neutrophil staining, I. Toludine blue for mast cell staining (arrows). Scale bar represents 200 μm.

**Supplement 2:**
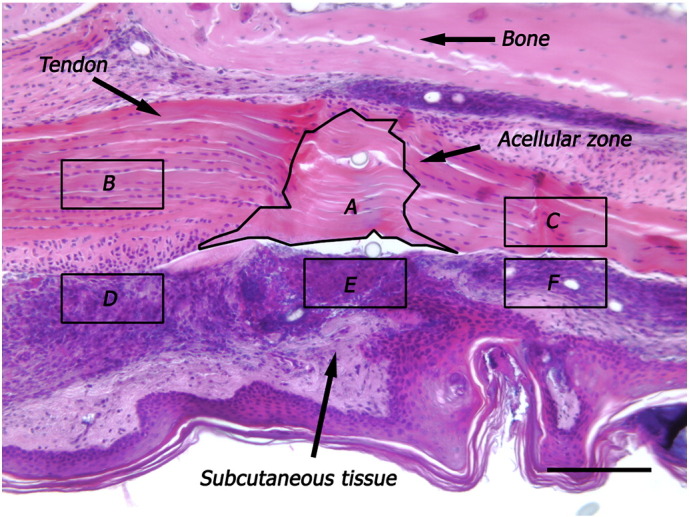
Sampling method for quantifying cellular expression. Pooled into tendon (A, B and C), and subcutaneous tissue (D, E, and F). Scale bar represents 200 μm.
